# An exploratory study with an adaptive continuous intravenous furosemide regimen in neonates treated with extracorporeal membrane oxygenation

**DOI:** 10.1186/cc6146

**Published:** 2007-10-10

**Authors:** Maria MJ van der Vorst, Jan den Hartigh, Enno Wildschut, Dick Tibboel, Jacobus Burggraaf

**Affiliations:** 1 Centre for Human Drug Research, Leiden, The Netherlands; 2 Department of Paediatric Surgery, Erasmus Medical Centre, Rotterdam, The Netherlands; 3 Department of Clinical Pharmacy and Toxicology, Leiden University Medical Center, Leiden, The Netherlands; 4 Department of Paediatrics, Erasmus Medical Centre, Rotterdam, The Netherlands

## Abstract

**Introduction:**

The objective of the present study was to explore a continuous intravenous furosemide regimen that adapts to urine output in neonates treated with extracorporeal membrane oxygenation (ECMO).

**Methods:**

Seven neonates admitted to a paediatric surgical intensive care unit for ECMO therapy were treated with a furosemide regimen consisting of a loading bolus (1–2 mg/kg) followed by a continuous infusion at 0.2 mg/kg per hour, which was adjusted according to the target urine production of 6 ml/kg per hour. Therapeutic drug monitoring for furosemide concentrations in blood was performed.

**Results:**

The mean ± standard deviation furosemide dose was 0.17 ± 0.06 mg/kg per hour, 0.08 ± 0.04 mg/kg per hour and 0.12 ± 0.07 mg/kg per hour, respectively, on the first day, second day and third day of the study. The median (range of the urine production of the study subjects) urine production over the consecutive study days was 6.8 (0.8–8.4) mg/kg per hour, 6.0 (4.7–8.9) mg/kg per hour and 5.4 (3.4–10.1) ml/kg per hour. The target urine production was reached after a median time of 7 (3–37) hours. The regimen was haemodynamically well tolerated and the median furosemide serum concentration was 3.1 (0.4–12.9) μg/ml, well below the toxic level.

**Conclusion:**

The evaluated furosemide infusion appears an effective means to reduce volume overload in neonates treated with ECMO. The data of this preliminary study suggest that the starting dose of furosemide was too high, however, because the urine output was excessive and required frequent adaptations. The results of this study therefore indicate that a novel pharmacokinetic/pharmacodynamic model needs to be developed for neonates treated with ECMO.

## Introduction

Extracorporeal membrane oxygenation (ECMO) is used mainly in neonates to treat a variety of cardiorespiratory problems such as meconium aspiration syndrome, congenital diaphragmatic hernia, persistent pulmonary hypertension of the newborn, and sepsis/pneumonia [1].

The ECMO circuit, like the cardiopulmonary bypass (CPB) circuit, triggers an important inflammatory reaction and is clinically associated with the so-called capillary leakage syndrome, resulting in intravascular hypovolaemia and renal hypoperfusion [2]. Consequently, in the initial phase (in the first 24–48 hours) the ECMO patient becomes usually increasingly oedematous. Diuretics, especially loop diuretics such as furosemide, are therefore the mainstay in the enhancement of diuresis to mobilize fluid excess. Furosemide is often used as a continuous infusion in patients treated with ECMO, based upon the observations in infants after CPB surgery [3-6].

We recently made an inventory of furosemide regimens used in neonates treated with ECMO and concluded that continuous intravenous furosemide was frequently used, but the used regimens varied widely in continuous doses and additional intermittent doses [7]. Although adequate urine output was achieved within 24 hours with all regimens, the used furosemide regimens might not be the optimal regimen for neonates treated with ECMO. In an accompanying editorial it was suggested that development of more standardized and efficacious dosing regimens would be preferable [8].

Since ECMO and CPB result in fluid overload, at least partially based on the same pathophysiology, it seems reasonable to assume that pharmacokinetic/pharmacodynamic (PK/PD) models developed for infants following cardiac surgery might also be applicable for neonates treated with ECMO [9]. We therefore conducted a prospective exploratory study in neonates treated with ECMO to evaluate a suggested furosemide regimen that was initially developed for infants after CPB surgery. The regimen consisted of a continuous furosemide infusion at a rate of 0.2 mg/kg per hour that was preceded by a loading bolus. The aim was to achieve a urine output of 6 ml/kg per hour. The main objectives of the study were to establish the efficacy of such a regimen and also to document serum furosemide concentrations to rule out ototoxic levels.

In the present article we report the findings of the proposed furosemide regimen in neonates treated with venoarterial ECMO in our unit.

## Materials and methods

The study was performed at the paediatric surgical intensive care unit of the Sophia Children's Hospital of Erasmus Medical Centre in Rotterdam, The Netherlands. The study protocol was approved by the Committee on Medical Ethics of the Erasmus Medical Centre and was conducted according to the principles of the Declaration of Helsinki. Parental written informed consent was obtained for all patients.

### Patients

Consecutive patients younger than 1 year of age who were admitted to our unit for ECMO treatment were enrolled in the study. Continuous intravenous furosemide was started when the patient was in a cardiovascular stable condition. The patient was considered cardiovascularly stable if there was no need for ongoing fluid resuscitation and/or for an increase in inotropic support. The amount of inotropic support was quantified by the vasopressor score [10,11].

Demographic and clinical data were collected from the patient charts and from the electronic patient data management system. This data included the gestational and postpartum age, gender, weight, diagnosis, the ECMO flow and duration of ECMO treatment, the time when continuous furosemide infusion was started, the doses and duration of continuous intravenous furosemide, additional loop diuretics, inotropic support, and fluid intake.

The following variables were measured before the study and at regular time intervals during the study for a maximum of 72 hours: urine output, heart rate, and mean arterial blood pressure. Serum albumin, creatinine, and BUN levels, and the arterial blood gas, were determined at regular intervals during the observation period.

Blood samples for the determination of serum furosemide concentrations were taken 10 minutes after the (loading) bolus dose, and additional samples were taken when possible. All patients had a urinary catheter as part of standard treatment according to the standard hospital ECMO protocol. The observation period for the study was 72 hours after the start of the continuous infusion. Serum electrolyte levels were closely monitored during the continuous intravenous furosemide therapy, and supplements were given if necessary.

### Furosemide regimen

The continuous furosemide infusion is started at a rate of 0.2 mg/kg per hour and is preceded by a loading bolus, the dose of which is dependent on renal function. Patients with normal renal function received 1 mg/kg and patients with acute renal failure received 2 mg/kg. Acute renal failure was defined on plasma creatinine levels and depended on the gestational and postpartum age [12].

The aim was to reach and maintain a urine output of 6 ml/kg per hour. Adaptation of the infusion rate was allowed when the target urine level was not reached at two consecutive hourly assessments. If the urine production was less than 4 ml/kg per hour, the rate of infusion could be increased; and if urine production was more than 8 ml/kg per hour, the infusion rate could be decreased.

### Sampling and assays

Routine blood samples were analysed at the Clinical Chemistry Laboratory of the Erasmus Medical Centre. Furosemide concentrations were measured using a validated high-performance liquid chromatography method routinely applied at the laboratory of Clinical Pharmacy and Toxicology of Leiden University Medical Center [6]. For determination in serum, the coefficient of variation of the assay at 1 μg/ml was 2%, and the reproducibility of the slope was 8.9%.

### Data analysis

Data showing a skewed distribution are presented as the median (range), while the normally distributed data are presented as the mean ± standard deviation. The outcome evaluation included the median urine production over each 24-hour time interval and the time at which the target urine production was reached. The time to attain the target urine production was defined as the time point at which urine production was at least 6 ml/kg per hour for two consecutive hourly assessments.

## Results

### General

Continuous intravenous furosemide was evaluated in seven patients in whom venoarterial ECMO was performed. The study population consisted of six female patients and one male patient. The median gestational age was 40 (26–41) weeks. On admission, the median postpartum age was 3 (0–136) days and the median weight was 3.8 (3.0–5.0) kg. ECMO was performed for meconium aspiration syndrome in three patients, for respiratory insufficiency in three patients, and for persistent pulmonary hypertension of the newborn in one patient. ECMO was started 2 (0–65) hours after admission. All patients were weaned from ECMO after 109 (47–272) hours and were discharged from the intensive care unit after 7 (4–33) days.

### Extracorporeal membrane oxygenation regimen

The priming volume of the ECMO circuit was approximately 400 ml, and the solution consisted of albumin and packed red blood cells. The initial median ECMO flow was 101 (59–132) ml/kg per minute, equal to 80% of the total cardiac output. The median ECMO flow at the start of the continuous furosemide therapy and after 8, 16, 24, 48, and 72 hours of continuous furosemide infusion were, respectively, 109 (59–139) ml/kg per minute, 102 (76–139) ml/kg per minute, 97 (67–167) ml/kg per minute, 125 (76–167) ml/kg per minute, 116 (52–153) ml/kg per minute, and 82 (40–139) ml/kg per minute.

### Furosemide regimen

Continuous furosemide infusion was started 3 (0–22) hours after the start of ECMO at a rate of 0.2 mg/kg per hour and was preceded by a loading bolus of 1 ± 0.04 mg/kg. The mean ± standard deviation furosemide dose was 0.17 ± 0.06 mg/kg per hour, 0.08 ± 0.04 mg/kg per hour, and 0.12 ± 0.07 mg/kg per hour, respectively, over the first day, second day, and third day of the study.

The dose needed to be decreased from the first to the second day in five out of the seven patients, indicating that the starting dose was too high. No additional furosemide boluses were administered during the continuous furosemide infusion. The total administered furosemide dose was 4.97 (2.70–7.02) mg/kg per 24 hours, 1.63 (0.75–4.31) mg/kg per 24 hours, and 1.50 (0.09–6.3) mg/kg per 24 hours on the three consecutive study days. The total administered furosemide dose over 72 hours was 7.0 (4.97–14.21) mg/kg. The furosemide regimen is depicted in Table 1.

**Table 1:** Furosemide regimen

Furosemide therapy	Before therapy	0–24 hours	24–48 hours	48–72 hours	0–72 hours	
Bolus intravenous furosemide						
◦ Patients (*n*)	7					
◦ Mean dose (mg/kg per hour)	1 ± 0.04					
Continuous intravenous furosemide						
◦ Patients (*n*)		7	6	5		
◦ Mean dose (mg/kg per hour)		0.17 ± 0.06	0.08 ± 0.04	0.14 ± 0.09		
Total intravenous furosemide						
◦ Patients (*n*)		7	6	5		
◦ Median dose (mg/kg per 24 hours)		4.97 (2.70–7.02)	1.24 (0–4.31)	1.60 (0.09–6.4)		
◦ Median dose (mg/kg per 72 hours)					7.00 (4.97–14.3)	

Data presented as the mean ± standard deviation or as the median (range).

The median duration of the continuous furosemide infusion during ECMO was 70 (19–276) hours, which is in accordance with 75% (37–100%) of the ECMO time. Continuous furosemide infusion was discontinued 23 (4–120) hours before decannulation in six patients, and in one patient it was discontinued 4 hours after decannulation.

### Furosemide pharmacokinetics

The apparent volume of distribution was 0.5 (0.2–2.7) l/kg. The furosemide concentration 10 minutes after the loading bolus was 1.95 (0.4–4.7) μg/ml, and the concentration in all of the samples (*n *= 15) taken during the entire observation period was 3.1 (0.4–12.9) μg/ml.

### Urine output and fluid balance

**Figure 1:**
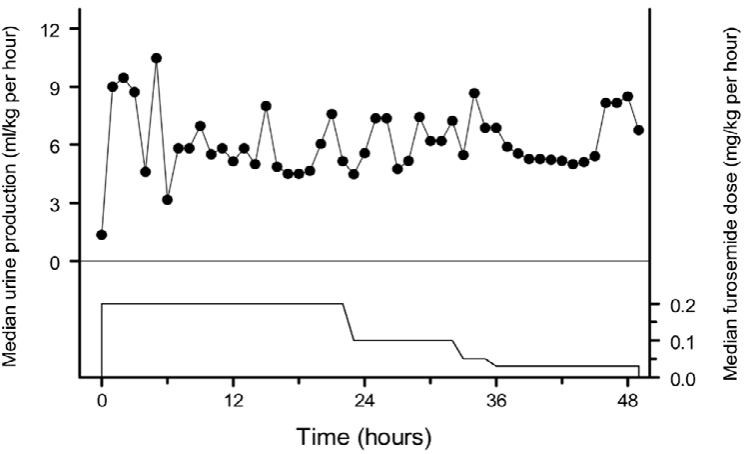
Overview of the median furosemide dose and urine production.

The overview of the median furosemide dose and urine production shows that the urine production first exceeds the target and is subsequently within the limits (Figure 1). Urine production from the start of ECMO until the start of furosemide therapy was 2.2 (0.7–9.6) ml/kg per hour, and increased to 7.9 (0.3–12.0) ml/kg per hour and 6.1 (0.2–9.2) ml/kg per hour after 8 and 16 hours, respectively, of continuous furosemide infusion. The median urine production over the consecutive study days was 6.8 (0.8–8.4) ml/kg per hour, 6.0 (4.7–8.9) ml/kg per hour, and 5.4 (3.4–10.1) ml/kg per hour. An overview of the median furosemide dose and urine production is depicted in Table 2.

**Table 2:** Median furosemide dose and urine production

Furosemide therapy time (hours)	Patients (*n*)	Furosemide dose (mg/kg per hour)	Urine production (ml/kg per hour)
0	7	0.20	2.2 (0.7–9.6)
8	7	0.20 (0.12–0.24)	7.9 (0.3–12.0)
16	7	0.20 (0.05–0.30)	6.1 (0.2–9.2)
24	7	0.19 (0.04–0.21)	4.7 (2.0–9.4)
32	6	0.10 (0.00–0.16)	6.6 (0.6–9.2)
40	6	0.07 (0.02–0.10)	6.4 (2.4–8.7)
48	6	0.08 (0.03–0.19)	5.8 (4.3–8.0)
56	5	0.10 (0.03–0.30)	6.5 (3.4–10.3)
64	4	0.08 (0.05–0.30)	3.9 (2.3–10.9)
72	4	0.10 (0.05–0.23)	4.8 (3.3–7.9)

Data presented as the median (range).

Over the entire study period the median urine production was 6.7 (4.1–8.8) ml/kg per hour, resulting in a median cumulative urine production of 369 (168–524) ml/kg.

The target urine production was reached after a median time of 7 (3–37) hours. Thereafter the median urine production remained at the target level of 6.0 ml/kg per hour.

Median fluid balances in the first 24 hours, calculated over 8-hour intervals, were -50.9 ml, +63.1 ml, and +82 ml, respectively. The median 24-hour balance over the three study days were, respectively, +3 (-267.9 to 624.1) ml, -4.6 (-202.0 to 397.3) ml, and +45 (-430.0 to 283.0) ml.

### Cardiovascular effects

The median mean arterial pressure and the heart rate at the start of ECMO were 48 (37–64) mmHg and 156 (112–170) beats/minute, and at the start of the furosemide treatment the respective values were 51 (37–73) mmHg and 146 (131–170) beats/minute. After 8, 16, 24, 48, and 72 hours of furosemide treatment, the median mean arterial pressure and heart rate were 49 (40–107) mmHg and 161 (136–173) beats/minute, 52 (39–93) mmHg and 155 (135–175) beats/minute, 52 (46–68) mmHg and 162 (145–181) beats/minute, 51 (50–65) mmHg and 153 (134–185) beats/minute, and 47 (46–48) mmHg and 152 (117–155) beats/minute, respectively. All cardiovascular parameters were within the normal range for age [13,14].

All patients remained cardiovascularly stable during the administration of continuous intravenous furosemide, and the inotropic support was gradually decreased during the observation period. The number of patients requiring inotropic support was decreased during the study from seven out of seven patients (100%) to two out of seven patients (29%). The median vasopressor score at start of ECMO was 20 (5–130), and that at the start of the continuous furosemide infusion was 15 (0–110). After 8, 16, 24, 48, and 72 hours of continuous furosemide treatment, the median vasopressor score was 15 (0–90), 10 (0–90), 20 (0–55), 20 (0–42), and 5 (0–10), respectively.

### Renal function

Median serum creatinine levels at the start of ECMO and at the start of continuous intravenous furosemide infusion were, respectively, 35 (19–106) μmol/l and 30 (19–106) μmol/l. After 24, 48, and 72 hours of continuous intravenous furosemide treatment, the median serum creatinine levels were 41 (16–131) μmol/l, 44 (22–112) μmol/l, and 23 (20–41) μmol/l, respectively. The median serum BUN level was 2.1 (1.1–3.8) mmol/l at the start of ECMO, and was 2.2 (1.1–3.8) mmol/l at the start of continuous intravenous furosemide. After 24, 48, and 72 hours of furosemide infusion, the median serum BUN levels were 3.7 (0.9–8.0) mmol/l, 6.0 (0.9–7.1) mmol/l, and 2.1 (1.5–6.0) mmol/l, respectively. The median serum albumin levels at the start of ECMO and at the start of furosemide infusion were 24 (19–27) g/l and 26 (23–35) g/l. During continuous intravenous furosemide treatment, the median serum albumin levels were 28 (25–34) g/l, 28 (25–31) g/l, and 29 (27–29) g/l after 24, 48, and 72 hours, respectively. The renal function is summarized in Table 3.

**Table 3:** Renal function and metabolic effects

	Start of ECMO	0 hours	24 hours	48 hours	72 hours
Renal function					
◦ Creatinine (μmol/l)	35 (19–106)	29.5 (19–106)	40.5 1(6–131)	44 (22–112)	23 (20–41)
◦ BUN (mmol/l)	2.05 (1.1–3.8)	2.2 (1.1–3.8)	3.7 (0.9–8)	6 (0.9–7.1)	2.1 (1.5–6)
◦ Albumin (g/l)	24 (19–27)	26 (23–35)	28 (25–34)	28 (25–31)	29 (27–29)
Acid-base balance					
◦ pH	7.3 (6.97–7.47)	7.4 (7.24–7.47)	7.42 (7.38–7.48)	7.48 (7.43–7.6)	7.47 (7.45–7.67)
◦ Bicarbonate level (mmol/l)	22.2 (17.4–33.5)	24.2 (17.4–33.5)	29.8 (23.4–35.2)	31.8 (23.8–35.1)	33.9 (26.3–36.5)
◦ Base excess	-4 (-12 to 9)	1 (-9 to 9)	5 (-1 to 10)	7 (1–10)	8 (3–14)
Serum electrolytes					
◦ Sodium (mmol/l)	140 (138–147)	142 (136–147)	136 (132–142)	135 (133–143)	134 (132–141)
◦ Potassium (mmol/l)	3.3 (3.1–4.1)	3.3 (2.8–5.4)	3.85 (3.2–6.2)	3.6 (3.1–4.1)	3.9 (3.5–5.7)
◦ Chloride (mmol/l)	106.5 (104–109)	104 (104–104)	102 (100–112)	95 (92–98)	99 (95–107)

Data presented as the median (range). ECMO, extracorporeal membrane oxygenation.

### Metabolic effects

Metabolic alkalosis, defined as pH > 7.45 and (actual) serum bicarbonate > 29 mmol/l, was observed in two patients after 48 hours of continuous furosemide infusion. The pH value, (actual) bicarbonate level, and base excess at the start of ECMO and during the continuous furosemide treatment are depicted in Table 3.

Serum electrolytes were within the normal range for age during the study (Table 3). Hypochloraemia (92 mmol/l) was observed in one patient with metabolic alkalosis.

## Discussion

Since the observation that continuous intravenous furosemide might be superior to intermittent administrations in infants after CPB surgery, the use of continuous furosemide infusion has increasingly be documented in patients following CPB surgery [3-6,15]. Based upon the observations in infants after CPB surgery, the use of continuous intravenous furosemide in neonates treated with ECMO is increasing.

We recently evaluated furosemide regimens used in neonates treated with ECMO in our unit and concluded that continuous intravenous furosemide was frequently used in neonates (78%) treated with ECMO [7]. The furosemide regimens used varied widely, in continuous doses and in additional intermittent doses. Although all used regimens achieved adequate urine output within 24 hours, the use of additional furosemide bolus injections suggests that the regimens might not be the optimal for neonates treated with ECMO, and therefore dosing regimens should be developed [7].

Since ECMO and CPB are 'comparable' procedures, the developed PK/PD model for infants after CPB surgery might also be applicable for neonates treated with ECMO [9]. There are, however, obvious differences between ECMO and CPB: in the time of exposure to the procedure, and thereby the presence of the 'circuit' with an ongoing inflammatory reaction, in the underlying illness and in the age of the patients. We therefore conducted a prospective exploratory study in neonates treated with ECMO to evaluate a suggested furosemide regimen developed for infants after CPB surgery. The results suggest that the used regimen was effective and well tolerated in neonates treated with ECMO.

Continuous intravenous furosemide was started in all patients at a rate of 0.2 mg/kg per hour and was preceded by a loading bolus of 1 mg/kg. The furosemide dose was adapted according to urine output. The dose was decreased from the first day to the second day of the study, from 0.17 ± 0.06 mg/kg per hour to 0.08 ± 0.04 mg/kg per hour. The furosemide doses used in neonates treated with ECMO (0.17 ± 0.06, 0.08 ± 0.04, and 0.12 ± 0.07 mg/kg per hour) were lower than the doses used in infants after CPB surgery (0.22 ± 0.06, 0.25 ± 0.10, and 0.22 ± 0.11 mg/kg per hour) over the first day, second day, and third day of furosemide therapy, respectively [16].

The PK/PD model for diuretic therapy with furosemide in infants after CPB suggested that doses between 0.2 and 0.3 mg/kg per hour, preceded by a loading bolus, would result in a urine production of 6 ml/kg per hour [9]. Based upon our observational study, which indicated that relatively low doses of continuous furosemide were used, we decided to use the lowest dose suggested by the model. The rational for the loading bolus was based on the simulated urine production profiles generated with the use of different furosemide regimens and on the observed effects of the loading bolus in the retrospective study [7,9].

In the retrospective study, positive effects of the 'loading' bolus were observed, although not statistically significant, in the urine output in the first 24 hours and in the time to reach the desired urine output of 6 ml/kg per hour [7]. Also, no additional furosemide bolus injections were administered during the continuous infusion to the patients who received a bolus prior to the continuous infusion. These observed effects might suggest that one loading bolus might be sufficient to overwhelm the effects of the ECMO circuit.

The data from the present study suggest that the starting dose was too high, as indicated by the urine output exceeding the target urine output in the first 24 hours. Although a full understanding of this phenomenon is hard to reach, it seems logical to assume that contributing factors might be the ECMO circuit, the renal function of the patients, and the age of the patients [17-23]. The patients treated with ECMO were younger (median 3 days) than the patients after CPB surgery (median 12 weeks), and therefore by definition had a less mature renal function, which leads to a decreased renal clearance of furosemide.

The renal function (median creatinine 30 μmol/l) was normal for age in the ECMO patients, whereas (transient) renal failure (median creatinine 95 μmol/l) was observed in the majority of the patients after CPB surgery [12,16]. Therefore it can be hypothesized that the acute renal failure observed in the patients after CPB surgery had a major impact on renal clearance, which is most closely related with drug response, since furosemide is excreted renally and only acts after reaching the tubular lumen [24-27]. This hypothesis might explain why higher doses were needed in the patients after CPB surgery. In addition, phase II reactions are better developed in infants and, as a result, the percentage of furosemide glucuronide will be higher [23]. Less unchanged furosemide can therefore be assumed available to interact with the furosemide receptor in the infants included in the cardiac surgery study, and higher doses are consequently needed to reach the same furosemide excretion rate [25,26]. This assumption might clarify why higher doses were required in the patients after CPB surgery.

On the other hand, the lower continuous furosemide doses after the loading bolus used in the ECMO patients might be explained by the effects of the ECMO circuit [17,18]. The observed increased volume of distribution in our patients was in accordance with the values reported in the literature [17]. Wells and colleagues reported that the steady-state volume of distribution and the elimination half-life of the loop diuretic, bumetanide, in term neonates treated with ECMO were increased compared with values in premature and term neonates without ECMO, while the plasma clearance was similar for both groups [17].

The increased volume of distribution is not only due to the addition of a large exogenous blood volume for priming of the circuit, but is also caused by the possible absorption of furosemide onto the ECMO circuit components [18,28]. Scala and coworkers performed an *in vitro *analysis to identify loss of furosemide in the ECMO circuit and observed a reduction of 63–87% in the serum furosemide concentration over a 4-hour period. The loss of drug was most pronounced in the first 30 minutes [29]. Since the continuous infusion was started at the time of the bolus injection, and as only furosemide samples were taken during the continuous infusion, we could not estimate the furosemide clearance in our patients.

Mehta and colleagues recently published research on the potential sequestration of drugs to the ECMO circuit. *In vivo *experiments showed that there was a significant drug loss in crystalloid-primed circuits as well as in blood-primed circuits. For instance, the loss of analgetics ranged from 17% for morphine to 87–100% for fentanyl depending on the type of circuit [30]. In addition, our own group described a decreased clearance of morphine during the first 10 days of ECMO in neonates and infants treated with venoarterial ECMO compared with patients after noncardiac major surgery [31,32].

The furosemide loading bolus especially seems to compensate for the increased volume of distribution. Since the effects of furosemide are dependent on renal function, the apparent need for lower continuous furosemide dose might be explained by the absence of impaired renal function, and consequently the increased renal clearance, in the patients on ECMO compared with the patients post CPB surgery [24].

We previously noticed that additional loop diuretics were needed in approximately 40% of the patients on ECMO therapy during the continuous furosemide infusion [7]. In the present study no additional loop diuretics were needed, demonstrating that furosemide monotherapy is highly effective, which is a considerable advantage.

The total administered furosemide dose in the current study was substantial higher on the first day (4.97 mg/kg per 24 hours) than the dose used in our retrospective study (1.92 mg/kg per 24 hours). The respective doses were slightly lower on the second day and third day (1.63 mg/kg per 24 hours and 1.50 mg/kg per 24 hours in the present study compared with 1.92 mg/kg per 24 hours and 2.0 mg/kg per 24 hours in the retrospective study). The cumulative furosemide doses over the three study days, however, were comparable between the two studies. The cumulative furosemide dose in the current study showed less variation in dose [7]. Importantly serum furosemide levels remained far below the commonly accepted safety level for ototoxicity (50 μg/ml) [33].

To obtain an acceptable fluid balance with a maintenance fluid of 120–140 ml/kg per 24 hours, the target urine production is set at 6 ml/kg per hour in our unit. In all patients studied, the target urine production of 6 ml/kg per hour was obtained a median 7 hours after the start of the continuous infusion. This is considerable faster than in our retrospective study in which the target urine production was reached in median 24 hours. The rapid attainment of the target urine may be explained by the initial higher infusion rate and the loading bolus.

The observed variability in urine output was small (4.1–8.8 ml/kg per hour) throughout the entire observation period – although it was striking that in one patient, despite administration of a high dose of furosemide, the urine output remained low, if not negligible, for a period of approximately 33 hours. We could not identify an obvious cause for this. In our retrospective study in which the patients received additional intermittent furosemide bolus injections, the variability in urine output was 0.7–16.1 ml/kg per hour during the study period. This is in accordance with studies in infants after CPB surgery, where less variance in urine output was observed with continuous administration compared with intermittent furosemide administration [3-5]. This suggests that strict protocols for diuretic therapies reduce variability in patients' response. It is probable that a tailored PK/PD model for furosemide therapy in neonates treated with ECMO may further optimize diuretic therapy for these critically ill neonates.

The obtained fluid balances were approximately zero for all three study days, although with substantial variability. The forced diuresis was well tolerated, as shown by the stable haemodynamic parameters and by the reduction of the vasopressor score.

Hypochloraemic metabolic alkalosis is a well-known side effect of furosemide therapy. A tendency for metabolic alkalosis was observed in two patients after approximately 48 hours of furosemide therapy. Since hypochloraemia was present in one patient, furosemide therapy was most probably the cause of the metabolic alkalosis. We have no explanation, however, for the metabolic alkalosis in the other patient, after contraction alkalosis and prerenal failure were excluded, and no increased use of inotropic drugs was present. This aspect should be recognized in the ongoing development and testing of a PK/PD model including more patients.

## Conclusion

The evaluated furosemide regimen of 0.2 mg/kg per hour preceded by a loading of 1 mg/kg is an effective means to obtain rapid and sufficient diuresis without cardiovascular instability in neonates treated with ECMO with a relatively low interpatient variability in urine production. The present exploratory study, however, suggests that for neonates on ECMO the proposed furosemide regimen as used in infants after CPB is using furosemide doses for the continuous infusion that are too high. A PK/PD model should therefore be developed for neonates on ECMO, identifying factors such as the circuit age, renal function and albumin that influence drug disposition during ECMO.

## Key messages

• The furosemide regimen, proposed by the PK/PD model developed for infants after CPB surgery, is too high for neonates treated with ECMO.

• A PK/PD model should be developed for neonates on ECMO, identifying the factors that influence drug disposition during ECMO.

## Abbreviations

CPB = cardiopulmonary bypass; ECMO = extracorporeal membrane oxygenation; PK/PD = pharmacokinetic/pharmacodynamic.

## Competing interests

The authors declare that they have no competing interests.

## Authors' contributions

MMJvdV and JB designed the study, evaluated the data, and wrote the manuscript. JdH analysed the furosemide samples, EW and DT were involved with patient management.
